# Assessment of *Hermetia illucens* pupal exuviae fermented by *Bacillus amyloliquefaciens* as functional feed additive for red feather native chickens

**DOI:** 10.1016/j.psj.2025.105584

**Published:** 2025-07-18

**Authors:** Che Lun Chang, Shen Chang Chang, Li Jen Lin, Jhih Siang Chang, Min Jung Lin, Tzu Tai Lee

**Affiliations:** a Department of Animal Science, National Chung Hsing University, Taichung 402, Taiwan; b Southern Region Branch, Taiwan Livestock Research Institute, Ministry of Agriculture, Pingtung 91201, Taiwan; c Department of Animal Science, National Chiayi University, Chiayi 600, Taiwan; d School of Chinese Medicine, College of Chinese Medicine, China Medical University, Taichung 40402, Taiwan; e Bachelor of Program in Scientific Agriculture, National Pingtung University of Science and Technology, Pingtung 91201, Taiwan; f The iEGG and Animal Biotechnology Center, National Chung Hsing University, Taichung, 402, Taiwan; g Smart Sustainable New Agriculture Research Center (SMARTer), Taichung 402, Taiwan

**Keywords:** *Bacillus amyloliquefaciens*, *Hermetia illucens*, Chitin, Chitooligosaccharides, Red feather native chicken

## Abstract

The purpose of this study was to evaluate black soldier fly pupal exuviae (BSFP) fermented by *Bacillus amyloliquefaciens* (FBSFP) as a functional feed additive for Red Feather Native chickens. Moreover, it investigated the effects of the feed additive on growth performance, blood parameters, intestinal morphology, and microbiota composition. These chickens were allocated into five groups and their feeds are as follows: basal diet (control), basal diet supplemented with 1 × 10^7^ CFU/kg *BA* (BA), 0.5 % BSFP (BSFP2), 0.25 % FBSFP (contains 5 × 10^6^ CFU/kg *BA*, FBSFP1), and 0.5 % FBSFP (contains 1 × 10^7^ CFU/kg *BA*, FBSFP2). The experiment lasted for 70 days. Although there were no differences in the growth performance (1-70 d) among the five groups, the count of lactic acid bacteria in the ileum increased significantly in all groups, while the coliform counts decreased significantly in BA group compared to the control group. FBSFP1 resulted in higher jejunal villi height and crypt depth ratio. All treatments decreased the serum MDA contents of chickens. However, the serum catalase and glutathione peroxidase activities of FBSFP2 were significantly higher than those of other groups. Supplementation with FBSFP could significantly increase serum tumor necrosis factor-α levels in chickens and significantly decreased the lightness (L*) of breast muscle, meanwhile, significantly reducing the cooking loss of thigh muscle at the 0.5 % supplementation. Except for the BSFP group, supplementation with both BA and FBSFP (0.25 % and 0.5 %) in the diets decreased the counts of coliform bacteria and indole concentration in the litter. In conclusion, FBSFP demonstrated great potential as a feed additive for chickens by improving the intestinal microflora, morphology, and antioxidant capacity of chickens, and offered positive effects on litter quality.

## Introduction

Animals are often exposed to stress due to high-density breeding or high temperatures. These factors make animals susceptible to respiratory or digestive infections due to pathogens, thus making them more prone to diseases (Nardone et al., 2010; Sánchez et al., 2017b). For a long time, antibiotics have been used as feed additives to prevent disease and improve the efficiency of livestock production. However, pathogenic bacteria may be potentially resistant to antibiotics, leading to concerns about drug-residues in livestock products. In recent years, antibiotics have been banned by the EU for use in animal rearing. Therefore, extensive research on effective substitutes is highly important (Chen et al., 2025; Popova, 2017).

Chitooligosaccharides (COS) are small polysaccharide molecules degraded from chitin or chitosan. The transformation of chitin and chitosan to COS is an efficient method to decrease viscosity and increase solubility, and may potentially enhancing the feasibility of COS as functional substances for a variety of biological activities, such as antibacterial, anti-tumor, anti-oxidation, and immune-promoting effects (Mengíbar et al., 2013; Sánchez et al., 2017a; Xing et al., 2017). In our previous study, we examined the black soldier fly pupal exuviae (BSFP), which contained a large amount of chitin, and was fermented and evaluated for their *in vitro* antibacterial activity and antioxidant capacity. After protease treatment and fermentation by *Bacillus amyloliquefaciens* (*BA*), COS content was measured as the fermentation product (FBSFP). FBSFP showed great antibacterial effects against *Escherichia coli* and possessed reducing power, DPPH radical-scavenging activity, and ferrous chelating capacity.

In Taiwan, Red Feather Native chickens have long been recognized for their body size and feed efficiency among colored broilers (Lee et al., 2022; Chen et al., 2020). However, the advantages of Red Feather Native chickens have declined due to low uniformity, poor feed conversion rate, high cost of feeding, along with the impact of fast food culture on traditional Chinese food culture (Chen et al., 2020). Therefore, the chicken industry urgently needs improvement in native chicken production approaches. With years of selective breeding, the growth rate of native chickens has improved, and the consumption habits have evolved (Chen et al., 2020), thus leading to the shortening of the rearing period of the Red Feather Native chickens, typically 8 to 10 weeks. However, few studies have explored the optimum feeding mode for Red Feather Native chickens (Lee et al., 2022; Chen et al., 2020).

In Taiwan, the population of Red Feather chickens is approximately 12.396 million, accounting for 26.65 % of total broiler production, making it one of the most important commercial chicken breeds. Recent studies have explored the potential of using black soldier fly larvae as alternative protein sources in poultry diets (Dabbou et al., 2025). In our previous research, we examined the black soldier fly pupal exuviae (BSFP), which contains high levels of chitin, and fermented and evaluated for their in vitro antibacterial activity and antioxidant capacity. After protease treatment and fermentation by *BA*, COS content was measured as the fermentation product (FBSFP). The results showed that FBSFP has great antibacterial effect against *E. coli* and possessed reducing power, DPPH radical-scavenging activity, and ferrous chelating capacity. Therefore, the present study aimed to develop FBSFP as a functional feed additive and evaluate its effects when supplemented in the diet of 10-week-old female Red Feather Native chickens. Parameters assessed included growth performance, blood biochemical indices, intestinal morphology, and gut microbiota composition.

## Materials and methods

### Preparation of fermented black soldier fly pupal exuviae (FBSFP)

**Fig. 1:**
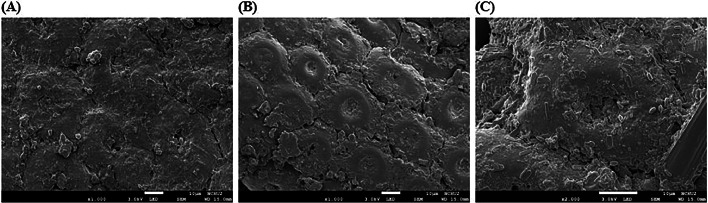
SEM photographs of BSFP (A), BSFP after protease treatment (B) and FBSFP (C) at 1000-2000 × magnification. FBSFP: BSFP fermented by BA after 3000U/g protease treatment.

The 5 g of BSFP and 0.3 g protease powder (50000 U/g) were added into a 250 mL Erlenmeyer flask covered with a double layer of aluminum foil (Fig. 1). After adding 50 mL RO water, the pH of the culture was adjusted to 7.2 and then incubated at 37 °C on a rotary shaker incubator at 125 rpm for 24 h. After protease treatment, 0.1 % K_2_PO_4_ and 0.05 % MgSO_4·_7H_2_O were added into the flask as the fermentation culture medium. The culture medium was sterilized by autoclaving at 121 °C for 30 min. *BA* inoculum was inoculated into the flask after cooling to room temperature. The *BA* used in this study were screened and selected by our laboratory (Teng et al., 2017; Tsai et al., 2021). The broth cultures were prepared by inoculating *BA* in Lysogeny broth at 37 °C for 24 h and the colony-forming units was verified achieving 1 × 10^7^ CFU/mL by plating serial 10-fold dilutions in duplicate into Lysogeny broth agar plates. The initial amount of *BA* was adjusted to 1 × 10^5^ CFU/mL and the culture medium was incubated at 37 °C on a rotary shaker incubator at 125 rpm for 5 d. After fermentation, the whole culture in the flask was mixed with the soybean meal and dried at 55 °C for 12 h for use in the Red Feather Native chicken feeding trial. The feeding program was divided into starter period (1–21 days), grower period (22–42 days), and finisher period (43–70 days). Feed intake was recorded daily. When the feed was nearly finished, additional feed was provided and the remaining amount was weighed to calculate the feed intake per pen. Diets were provided in mash form and water was provided *ad libitum* throughout the entire 70-day experimental period.

The reducing sugar content of culture supernatant was determined following the method of Gusakov et al. (2011). The cellulase activity of culture supernatant was determined based on the method of Johnsen and Krause (2014). The chitinase activity of culture supernatant was determined based on the methods (Wang et al., 2011).

### Experimental birds and housing

Four hundred 1-day-old female Red Feather Native chickens were randomly assigned to five treatment groups with four replicate pens per treatment and 20 birds per pen (totaling 80 birds per treatment). The initial average body weight (BW) was similar among different pens, with the average being approximately 37.5 to 38.5 g/bird. All the chickens were housed in pens sized 2 × 4 m^2^ and the rice hulls were spread as litter in a temperature-controlled environment. The temperature of the chicken house was maintained at 33 ± 1°C until they reached 7 d of age, then gradually decreased to 26 ± 1°C until the birds were 21 d old, after which, the temperature was maintained at 26 ± 1°C. During the entire rearing stage, there was 23-hour lighting and 1 hour of darkness per day.

### Dietary treatment and feeding schedule

Birds were fed a maize-soybean meal basal diet and the five treatments were fed as follows: basal diet (control), basal diet supplemented with 10^7^ CFU/kg *BA* (BA), basal diet supplemented with 0.5 % BSFP (BSFP2), basal diet supplemented with 0.25 % FBSFP (5 × 10^6^ CFU/kg *BA*, FBSFP1), and basal diet supplemented with 0.5 % FBSFP (1 × 10^7^ CFU/kg *BA*, FBSFP2). Nutritional ingredients and the chemical composition of the basal diet are listed in Table 1. The experiment lasted for 70 d and was divided into three phases: starter (1-21 d), grower (22-42 d), and finisher (43-70 d). The diets were provided in mash form and water was provided *ad libitum* during the entire experimental period (70 d). The diets were formulated to meet the requirements recommended by the National Research Council (1994).

**Table 1:** Ingredients and chemical composition of the experimental diets for Red Feather Native chickens.

Ingredients		Starter diet (1–21 days)	Grower diet (22–42 days)	Finisher diet (43–70 days)
		g/kg		
Corn, yellow		555	586	601
Soybean meal (CP-44 %)		295	260	230
Full fat soybean meal (CP-35.5 %)		50	75	100
Fish meal (CP-65 %)		50	25	12.5
Soybean oil		18	21	24
Monocalcium phosphate		13	14	14
Calcium carbonate		11.5	11	10.5
_DL_-Methionine		2.0	1.6	1.5
NaCl		2.5	3.0	3.5
Choline-Cl		1.0	1.0	1.0
Vitamin premix^1^		1.0	1.0	1.0
Mineral premix^2^		1.0	1.0	1.0
Total		1000.0	1000.0	1000.0
Composition	Calculated nutrient value			
ME, kcal/ kg		3025	3075	3125
Crude protein, %		22	20	19
Calcium, %		1.00	0.90	0.85
Total Phosphorus, %		0.64	0.60	0.56
Available Phosphorus, %		0.50	0.45	0.20
Lysine, %		1.29	1.13	1.04
Methionine, %		0.60	0.51	0.48
Methionine + Cystein, %		0.95	0.85	0.80
Threonine, %		0.86	0.78	0.73
Cl, %		0.21	0.23	0.25
Na, %		0.17	0.17	0.17
K, %		0.89	0.85	0.83

1 Supplied per kg of diet: Vit A 15000 IU; Vit. D3 3000 IU; Vit. E 30 mg; Vit. K3 4 mg; Riboflavin 8 mg; Pyridoxine 5 mg; Vit. B12 25 μg; Ca-pantothenate 19 mg; Niacin 50 mg; Folic acid 1.5 mg; Biotin 60 μg.

2 Supplied per kg of diet: Co (CoCO_3_) 0.255 mg; Cu (CuSO_4_∙5H_2_O) 10.8 mg; Fe (FeSO_4_∙H_2_O) 90 mg; Zn (ZnO) 68.4 mg; Mn (MnSO_4_∙H_2_O) 90 mg; Se (Na_2_SeO_3_) 0.18 mg.

### Performance and sample collection

During the experiment, the Red-feather native chickens are generally marketed at 10 weeks of age. Body weights were recorded at hatching, 8 weeks, and 10 weeks of age. Feed was provided *ad libitum*, and the amount of feed added was recorded each time. At the end of the experiment, feed intake for each stage was calculated accordingly. Body weight gain (BWG) and feed conversion ratio (FCR) were also calculated considering BW and FI. On day 70, eight chickens (two birds per pen) near the average weight were sacrificed for each treatment. A venous puncture was made using a vacuum blood collection tube. The blood sample was allowed to clot at room temperature for 3 h, centrifuged at 3000 x g for 30 min, and the serum was withdrawn. The serum sample was stored at −20°C, and subsequently, serum characteristics were evaluated. After blood collection, the birds were euthanized by electric shock, and the ileum and cecum were collected after dissection. The ileal and cecal contents were collected in sterile petri dishes for subsequent studies.

### Determination of intestinal microbial population

One gram of ileum or cecum contents was serially diluted with 9 mL of 0.9 % sterile saline and mixed well by vortexing. The samples with appropriately diluted and then injected in triplicate to chromogenic medium agar (CHROMagarTM 129 ECC), Tryptose Sulfite Cycloserine Agar, Perfringens Agar (TSC agar) and MRS medium (de Man Rogosa and Sharpe agar, Difco 288130, BD, Franklin Lakes, NJ) plates to isolate *E. coli*, coliform, and lactic acid bacteria, respectively. The chromogenic medium and MRS medium plates were incubated at 37°C for 48 h, and the TSC agar plates were incubated at 37°C for 48 h under anaerobic conditions. Microbial colonies were counted immediately after removing the plates from the incubator. The results were expressed as log CFU/g of intestinal contents (Chuang et al., 2021).

### Morphometric analysis of ileum and jejunum

At the end of the experiment (70 d), a single bird from each pen (four birds per treatment) was randomly selected and slaughtered by cutting their jugular vein. The intestinal tract was pulled out and the middle section of the intestinal ileum (from Michael's diverticulum to the ileum isthmus) was taken out. For subsequent morphometric analyses, a 2 cm long section of ileum and cecum was fixed in formalin. The formalin-fixed ileal and cecal sections were embedded in paraffin wax before washed with phosphate-buffered saline (PBS). Tissue sections (3 μm) were stained using hematoxylin and eosin staining methods. The samples were analyzed by optical microscopy and the villus height and crypt depth in 15 representative samples of each treatment were determined using a software (Motic image plus 2.0). The ratio of villus height to crypt depth was also calculated (Chuang et al., 2021).

### Serum antioxidant enzyme activity

The levels of malondialdehyde (MDA), superoxidase dismutase (SOD) activity, catalase (CAT) activity, and glutathione peroxidase (GSH-Px) activity were measured using specific assay kits (Cayman Chemical Co., Ltd.). Serum samples from each treatment were measured in triplicate and appropriate dilutions were made to allow the concentrations to fall within the range of standard curves.

### Serum characteristics

Serum samples were sent to the Health Medical Laboratory (Yunlin, Taiwan) for the analysis of serum characteristics. Serum biochemical indicators included serum glucose (GLU), urea nitrogen (BUN), creatinine (CREA), uric acid (UA), glutamic-oxaloacetic transaminase (SGOT), serum glutamic-pyruvic transaminase (SGPT), alkaline phosphatase (Alk-P), cholesterol (CHO), triglyceride (TG), high-density lipoprotein cholesterol (HDLC) and low-density lipoprotein cholesterol (LDLC). Chicken tumor necrosis factor α (TNF-α) content of serum was analyzed using kits purchased from Cusabio Biotech Co., Ltd. All indicators were measured in triplicates of serum samples.

### Meat quality and slaughter characteristics

Three chickens per pen were randomly selected and sacrificed after 12 h of water and feed deprivation. Immediately after depilation and the viscera removal, the liver, spleen, and abdominal fat were weighed. The slaughter rate was determined by the ratio between carcass weight and live weight. For evaluating the meat quality, the medial portion of breast and thigh muscles was examined for meat color (L*, lightness; a*, redness; b*, yellowness) by using a hand-held colorimeter. The pH value of breast meat and thigh meat was determined by a pH meter. Within 1 h after dissection, the pectoral muscles were weighed and placed on an aluminum baking tray. Samples were weighed before and after cooking for determining cooking loss percentage (Wattanachant et al., 2005).

### Litter characteristics

Litter samples were collected from five different fields in each pen in a clean plastic bag. After air extrusion, the bags were placed at room temperature for 3 h. The ammonia concentration of the air inside each bag was measured by a Multi-Gas Detector (BW Technologies Ltd, New York, USA), and the results were expressed as ppm. The 1 g of each litter sample was serially diluted with 9 mL of PBS (pH 7.2) and mixed well by vortexing. Bacterial counts in the samples were then measured by plating serial 10-fold dilutions in triplicate in chromogenic medium agar (CHROMagarTM 129 ECC) to isolate coliform*.* The plates were then incubated for 48 h at 37 °C. Microbial colonies were counted immediately after being removed from the incubator. The results were expressed as log colony-forming units (CFU) per gram of intestinal content. Indole and skatole concentrations were measured by gas chromatography (GC) according to the method of Larreta et al. (2008). Each litter sample (5 g) was mixed with 2.5 mL ether and 2.0 mL HCl in a 50 mL centrifuge tube. The tube was covered with paraffin and placed at −20 °C for 24 h. The supernatant was drained and filtered through 0.2 μm filtration film (Minisart, SRP 15 single-use syringe filter, Sartorius, Gottingen, Germany) in a microfuge tube (Eppendorf), and was stored at −20 °C before being analyzed. A 0.1 μL volume of filtered supernatant was injected into the GC (Clarus 580GC, PerkinElmer) coupled to a flame ionization detector (FID) (Hewlett-Packard, Little Falls, DE, USA) for the analysis. The temperature of the injection port was 250 °C and the split ratio was 14:1. The samples were separated on an HP5 MS (Agilent) capillary column (30 *m* × 0.25 mm × 0.25 μm) using the following program: 35 °C for 1 min, increase to 210 °C at 5 °C/ min and finally to 300 °C at 20 °C/ min. The carrier gas was N_2_ (99.995 %), which was kept constant at 10 psi pressure. H_2_ and air flow rate was set at 30 mL/min and 400 mL/min, respectively, and were used for FID detection. The peak areas of phenol and skatole of standard solutions and samples were integrated. The ratios of the peak areas of samples and standards were used to determine the concentration of phenol and skatole.

### Statistical analysis

The data were statistically analyzed using the general linear model (GLM) procedure of SAS software (2014) following a random arrangement. PROC UNIVARIATE was with the normal statement on the data to perform various normality tests.

The mathematic model used was:

$$Y_{ij}=\mu +T_{i}+\varepsilon_{ij}$$

where $Y_{ij}$ is the observed the response of the bird in the pen; µ is the overall mean; $T_{i}$ is the fixed effect of dietary treatment; and $\varepsilon_{ij}$ is the residual error when the pen was regarded as an experimental unit, $\varepsilon_{ij}∼N\left(0,\sigma_{\varepsilon }^{2}\right)$. The experimental unit was analyzed based on the five groups. Chickens were randomly distributed in 20 pens depending on a completely random design (*n* = 4), each pen contained 20 birds. All analyses were examined for significance through analysis of variance. The significance and mean separation were determined by Tukey’s honest significant difference test (HSD) using the SAS software program. Values were considered significant at *P* < 0.05 if the variables were significantly influenced by the treatments.

## Results

### Growth performance

The effects of *BA*, BSFP, and FBSFP supplementation on different phases of the growth performance of Red Feather Native chickens are shown in Table 2. From 1-56 d, 57-70 d, and 1-70 d of age, the differences in growth performance among all treatment groups were not significant (*P* > 0.05).

**Table 2:** Effects of *BA*, BSFP and FBSFP supplementation on growth performance of 1-70 d-old Red Feather Native chickens.

Item	Experimental diets					SEM	*P* value
	Control	BA	BSFP2	FBSFP1	FBSFP2		
1-56 d							
Body weight (g)^1^	1696	1686	1718	1730	1762	13.2	0.438
Feed consumption (g)^2^	3877	3902	3950	3894	3899	28.7	0.939
Weight gain (g)^2^	1660	1647	1681	1693	1725	13.2	0.421
FCR 2	2.34	2.37	2.35	2.30	2.26	0.02	0.591
57-70 d							
Body weight (g)^1^	1996	2005	2025	2048	2124	23.1	0.483
Feed consumption (g)^2^	800	835	810	790	838	35.0	0.990
Weight gain (g)^2^	301	348	307	318	362	15.4	0.666
FCR 2	2.66	2.40	2.64	2.49	2.31	0.10	0.826
1-70 d							
Feed consumption (g)^1^	4677	4737	4761	4684	4737	49.8	0.976
Weight gain (g)^2^	1960	1994	1988	2011	2087	23.1	0.487
FCR^2^	2.39	2.38	2.39	2.33	2.27	0.02	0.472

Control: basal diet; BA: basal diet supplemented with 10^7^ CFU/kg *BA.* BSFP2: basal diet supplemented with 0.5 % BSFP; FBSFP1: basal diet supplemented with 0.25 % FBSFP; FBSFP2: basal diet supplemented with 0.5 % FBSFP.

FCR = feed conversion ratio.

1 Each value represents the mean of 80 replicates.

2 Each value represents the mean of 4 replicates (20 birds in each replicate).

### The composition of protease activity fermentation on BSFP

The protease activity of 2000 U/g BSFP resulted in a higher reducing sugar content (11.2 mg/mL) compared to the 2000 U/g BSFP control and the untreated BSFP group (2.1 and 0.8 mg/mL, respectively). In addition to protease, the fermentation supernatant from the 2000 U/g protease treatment also exhibited higher cellulase and chitinase activities (3.0 U/mL vs. 1.1 U/mL and not determined, respectively; 22.0 mU/mL vs. 14.0 mU/mL and not determined, respectively).

### Intestinal microbial population

The effects of *BA*, BSFP, and FBSFP supplementation on microbial population in the ileum and cecum of Red Feather Native chickens after 70 d are shown in Table 3. Compared to the control group, the lactic acid bacteria count in the ileum increased significantly in all treatment groups (*P* < 0.05). However, the coliform count in the ileum decreased significantly in the *BA* treatment group compared to the control group (*P* < 0.05). There were no significant differences in *Clostridium perfringens* counts in ileum or cecum among all treatment groups (*P* > 0.05).

**Table 3:** Effects of *BA*, BSFP and FBSFP supplementation on intestinal microflora concentration of 70 d-old Red Feather Native chickens^1^.

Item	Experimental diets					SEM	*P* value
	Control	BA	BSFP2	FBSFP1	FBSFP2		
Lactic acid bacteria	Log CFU/g						
Ileum	7.73^b^	8.34^a^	7.95^b^	8.49^a^	8.53^a^	0.08	0.029
Ceca	8.44	8.46	8.39	8.44	8.55	0.15	0.887
Coliform	Log CFU/g						
Ileum	7.98^a^	7.41^b^	7.69^ab^	8.08^a^	7.91^ab^	0.09	0.032
Ceca	8.44	8.27	8.19	7.94	7.93	0.20	0.394
*Clostridium perfringens*	Log CFU/g						
Ileum	7.56	7.56	7.32	7.49	7.56	0.06	0.681
Ceca	8.19	7.93	8.33	8.18	7.98	0.06	0.289

Control: basal diet; BA: basal diet supplemented with 10^7^ CFU/kg *BA.* BSFP2: basal diet supplemented with 0.5 % BSFP; FBSFP1: basal diet supplemented with 0.25 % FBSFP; FBSFP2: basal diet supplemented with 0.5 % FBSFP.

^a,b^Means with in the same row with different letters are significantly different (*P* < 0.05).

1 Each value represents the mean of 4 replicates with 3 birds in each replicate.

### Intestinal morphology

The effects of *BA*, BSFP, and FBSFP supplementation on the intestinal morphology of 70-day-old Red Feather Native chickens are shown in Table 4. The differences in villi height and crypt depth in the jejunum or ileum among all treatment groups were not significant (*P* > 0.05). However, FBSFP supplementation (0.25 % groups) resulted in higher villus height and crypt depth ratio among the treatment groups compared to the control group (*P* < 0.05).

**Table 4:** Effect of *BA*, BSFP and FBSFP supplementation on intestinal morphology of 70 d-old Red Feather Native chickens^1^.

Item	Experimental diets					SEM	*P* value
	Control	BA	BSFP2	FBSFP1	FBSFP2		
Jejunum							
Villus height (μm)	913	1034	913	990	1009	20.3	0.227
Crypt depth (μm)	206	200	200	173	188	5.15	0.317
Villus height/Crypt depth	4.49^b^	5.16^ab^	4.63^ab^	5.86^a^	5.37^ab^	0.19	0.049
Ileum							
Villus height (μm)	842	928	848	873	887	16.2	0.452
Crypt depth (μm)	180	181	180	162	178	4.64	0.641
Villus height/Crypt depth	4.73	5.18	4.77	5.45	5.05	0.19	0.723

Control: basal diet; BA: basal diet supplemented with 10^7^ CFU/kg *BA.* BSFP2: basal diet supplemented with 0.5 % BSFP; FBSFP1: basal diet supplemented with 0.25 % FBSFP; FBSFP2: basal diet supplemented with 0.5 % FBSFP.

^a,b^Means with in the same row with different letters are significantly different (*P* < 0.05).

1 Each value represents the means of fifteen spots corresponding to four birds for each treatment.

### Serum antioxidant activity assessment

Table 5 shows the effects of *BA*, BSFP, and FBSFP supplementation in 70-day-old Red Feather Native chickens in terms of serum MDA concentration, SOD activity, CAT activity, and GSH-Px activity. Although the serum SOD activity did not differ among all treatments, there was a significant reduction in serum MDA contents of chickens fed with a diet containing either *BA*, BSFP, or FBSFP supplementation (*P* < 0.05). In addition, the serum CAT activity and GSH-Px activity of 0.5 % in FBSFP supplementation groups was significantly higher than those in other treatment groups (*P* < 0.05).

**Table 5:** Effects of *BA*, BSFP and FBSFP supplementation on serum antioxidant capacity of 70 d-old Red Feather Native chickens^1^.

Item	Experimental diets					SEM	*P* value
	Control	BA	BSFP2	FBSFP1	FBSFP2		
MDA (μM /ml)	32.1^a^	18.0^b^	21.4^b^	13.9^b^	15.2^b^	1.08	<0.001
SOD (U/mL)	37.8	40.2	40.6	34.7	41.6	1.30	0.460
CAT (U/mL)	377^b^	385^b^	374^b^	404^b^	526^a^	9.80	<0.001
GSH-Px (U/mL)	74.3^b^	70.5^b^	74.5^b^	79.5^b^	122^a^	3.51	<0.001

Control: basal diet; BA: basal diet supplemented with 10^7^ CFU/kg *BA.* BSFP2: basal diet supplemented with 0.5 % BSFP; FBSFP1: basal diet supplemented with 0.25 % FBSFP; FBSFP2: basal diet supplemented with 0.5 % FBSFP.

^a,b^Means with in the same row with different letters are significantly different (*P* < 0.05).

1 Each value represents the mean of four replicates (*n* = 4).

### Serum characteristics

The effects of *BA*, BSFP, and FBSFP supplementation on serum characteristics of 70-day-old Red Feather Native chickens are shown in Table 6. Compared to the control group, supplementation with 0.25 % and 0.5 % FBSFP significantly increased serum TNF-α (*P* < 0.05). In addition, supplementing *BA* in the diet significantly decreased serum CHOL compared to its level in the control group (*P* < 0.05). There were no significant differences in other items among all treatments (*P* > 0.05).

**Table 6:** Effect of *BA*, BSFP and FBSFP supplementation on serum characteristics of 70 d-old Red Feather Native chickens^1^.

Item	Experimental diets					SEM	*P* value
	Control	BA	BSFP2	FBSFP1	FBSFP2		
	mg/dL						
GLU	182	191	207	211	190	5.13	0.341
BUN	1.75	1.33	1.75	1.50	2.25	0.17	0.556
CREA	0.15	0.13	0.18	0.21	0.19	0.01	0.248
UA	5.98	5.33	4.95	5.80	5.25	0.40	0.917
	U/L						
SGOT	156	146	153	152	153	3.68	0.652
SGPT	2.75	2.33	3.25	2.00	2.25	0.42	0.883
	IU/L						
Alk-P	700	686	704	643	749	26.7	0.790
	mg/dL						
CHOL	109^a^	79^b^	112^a^	99^ab^	107^a^	3.21	0.021
TG	76.0	53.0	61.5	77.8	58.8	3.78	0.227
HDLC	61.8	54.7	66.5	62.0	57.3	2.06	0.147
LDLC	33.0	25.3	35.8	29.8	35.3	1.62	0.141
	pg/mL						
TNF-α	28.8^c^	32.9^c^	38.4^bc^	60.0^a^	52.2^ab^	2.53	0.007

GLU: glucose; BUN: urea nitrogen; CREA: creatinine; UA: uric acid; SGOT: glutamic-oxalocetic transaminase, SGPT: serum glutamic-pyruvic transaminase; TP: total protein, ALB: albumin; GLO: globulin; Alk-P: alkaline phosphatase; CHO: cholesterol; TG: triglyceride; HDL: high-density lipoprotein; LDL: low-density lipoprotein; TNF-α: chicken tumor necrosis factor α.

Control: basal diet; BA: basal diet supplemented with 10^7^ CFU/kg *BA.* BSFP2: basal diet supplemented with 0.5 % BSFP; FBSFP1: basal diet supplemented with 0.25 % FBSFP; FBSFP2: basal diet supplemented with 0.5 % FBSFP.

^a-c^ Means with in the same row with different letters are significantly different (*P* < 0.05).

1 Each value represents the mean of four replicates (*n* = 4).

### Carcass characteristics and organ weight

The effects of *BA*, BSFP, and FBSFP supplementation on carcass characteristics and organ weight of 70-day-old Red Feather Native chickens are shown in Table 7. The carcass weight, slaughter rate, kidney weight, spleen weight, and abdominal weight among all treatment groups did not differ significantly (*P* > 0.05).

**Table 7:** Effects of *BA*, BSFP and FBSFP supplementation on carcass characteristics and organ weight of 70 d-old Red Feather Native chickens^1^.

Item	Experimental diets					SEM	*P* value
	Control	BA	BSFP2	FBSFP1	FBSFP2		
Live weight (g)	1975	2015	2025	1943	2097	27.0	0.467
Carcass weight (g)	1786	1773	1765	1700	1919	31.5	0.297
Slaughter rate (%)	90.3	88.1	87.1	86.5	91.4	0.81	0.390
Kidney (%)	2.19	2.28	2.78	2.74	2.51	0.06	0.681
Spleen (%)	0.33	0.27	0.34	0.31	0.33	0.06	0.289
Abdominal fat (%)	2.10	2.39	2.64	2.00	2.39	0.10	0.277

Control: basal diet; BA: basal diet supplemented with 10^7^ CFU/kg *BA.* BSFP2: basal diet supplemented with 0.5 % BSFP; FBSFP1: basal diet supplemented with 0.25 % FBSFP; FBSFP2: basal diet supplemented with 0.5 % FBSFP.

1 Each value represents the mean of 4 replicates (*n* = 4).

### Meat quality

The effects of *BA*, BSFP, and FBSFP supplementation on the meat quality of 70-day-old Red Feather Native chickens are shown in Table 8. The meat quality was assessed separately for breast and thigh muscles. In the breast muscle, supplementation with 0.25 % FBSFP and 0.5 % FBSFP in diet significantly decreased L* compared to its value in the control group (*P* > 0.05). In addition, supplementation with *BA* and 0.5 % FBSFP significantly increased the b* value of breast muscle (*P* < 0.05) compared to its value in the control and BSFP2 groups. Evaluation of thigh muscle meat quality revealed no differences in L*, a*, b*, and pH values among all treatment groups (*P* > 0.05). Nevertheless, supplementation with 0.5 % FBSFP in the diet significantly decreased cooking loss of thigh muscle compared to the control group (*P* < 0.05).

**Table 8:** Effect of *BA*, BSFP and FBSFP supplementation on meat quality of 70 d-old Red Feather Native chickens^1^.

Item	Experimental diets					SEM	*P* value	
	Control	BA	BSFP2	FBSFP1	FBSFP2			
Breast muscle								
L* (lightness)	62.8^ab^	62.8^ab^	65.3^a^	61.6^c^	59.5^c^	2.07	0.001	
a* (redness)	5.69	5.44	4.38	5.47	5.19	1.37	0.522	
b* (yellowness)	14.4^b^	17.2^a^	14.6^b^	15.9^ab^	16.8^a^	1.72	0.029	
pH	5.63	5.58	5.74	5.66	5.56	0.16	0.128	
Cooking loss (%)	17.1	17.3	16.2	17.0	14.4	0.35	0.359	
Thigh muscle								
L* (lightness)	54.2	58.3	54.6	56.3	57.2	0.58	0.157	
a* (redness)	10.0	8.95	10.6	9.13	8.57	0.37	0.375	
b* (yellowness)	13.6	12.5	13.9	14.4	13.8	0.34	0.496	
pH	6.22	6.20	6.13	6.16	6.15	0.23	0.229	
Cooking loss (%)	18.1^a^	12.7^ab^	13.0^ab^	21.8^a^	12.0^b^	0.91	0.034	

Control: basal diet; BA: basal diet supplemented with 10^7^ CFU/kg *BA.* BSFP2: basal diet supplemented with 0.5 % BSFP; FBSFP1: basal diet supplemented with 0.25 % FBSFP; FBSFP2: basal diet supplemented with 0.5 % FBSFP.

^a-c^ Means with in the same row with different letters are significantly different (*P* < 0.05).

1 Each value represents the mean of four replicates (*n* = 4).

### Litter characteristics

The effects of *BA*, BSFP, and FBSFP supplementation on meat quality of litter characteristics are shown in Table 9. Counts of coliform bacteria, ammonia concentration, indole concentration, and skatole concentration of litters were analyzed for each group. A significant reduction in litter odor was noted in the *BA*, FBSFP1, and FBSFP2 groups. Except for the BSFP supplementation group, supplementation of *BA* and FBSFP (0.25 % and 0.5 %) in diet decreased the counts of coliform bacteria and indole concentration in litter (*P* < 0.05). The *BA* group showed a decreased skatole concentration in litter compared to the control group (*P* < 0.05).

**Table 9:** Effects of *BA*, BSFP and FBSFP supplementation on litter characteristics^1^.

Item	Experimental diets					SEM	*P* value
	Control	BA	BSFP2	FBSFP1	FBSFP2		
*E.coli* (LogCFU/ g)	5.55^a^	4.65^bc^	5.11^ab^	4.36^c^	4.34^c^	1.74	0.001
Ammonia (ppm)	114^a^	99^bc^	109^ab^	100^bc^	90^c^	0.08	0.007
Indole (ppm)	10.1^a^	6.06^b^	7.86^ab^	4.58^b^	6.60^b^	0.45	0.016
Skatole (ppm)	11.7^a^	5.32^b^	8.83^ab^	7.88^ab^	8.93^ab^	0.71	0.018

Control: basal diet; BA: basal diet supplemented with 10^7^ CFU/kg *BA.* BSFP2: basal diet supplemented with 0.5 % BSFP; FBSFP1: basal diet supplemented with 0.25 % FBSFP; FBSFP2: basal diet supplemented with 0.5 % FBSFP.

^a,b,c^Means with in the same row with different letters are significantly different (*P* < 0.05).

1 Each value represents the mean of four replicates (*n* = 4).

## Discussion

Past studies on the effects of *BA* on broiler chickens found that supplementation of *BA* in diet could improve the daily weight gain and FCR of broilers (Chen et al., 2025; LTsai et al., 2022; Ahmed et al., 2014; Lei et al., 2014, 2015). *BA* can stimulate the secretion of extracellular enzymes, including α-amylases, cellulase, metalloproteases, and proteases, thereby enhancing digestion and absorption, promoting intestinal health, and improving growth performance (Jha et al., 2020). *BA* possesses immunomodulatory functions, enhancing T cell activation and proliferation, and contributing to the prevention and reduction of antimicrobial use in chickens (Larsberg et al., 2023). Studies have shown growth-promoting effects as the number of *BA* supplemented in diets exceeded 10^8^ CFU/kg (Ahmed et al., 2014; Lei et al., 2014, 2015). In this study, supplementation of BSFP (0.5 %) and FBSFP (0.25 % and 0.5 %) did not show any negative impact on growth performance (Table 2). In contrast with the earlier results, including the treatment of *BA* supplementation, there was no significant growth promoting effect in this study. This inconsistency in the findings is possibly due to the difference in animal species and the number of *BA* supplemented in diets.

Complex carbohydrates in the diet, such as fibers, are not easily metabolized by lactic acid bacteria (Hammes and Hertel, 2006). However, the fiber-degrading enzymes such as cellulase and xylanase produced by *BA* during digestion in chickens, might degrade fibers into low-molecular-weight carbohydrates, thereby, leading to the increased lactic acid bacterial count in the ileum of chickens. A similar inhibitory effect on *E. coli* was observed in the intestine of broilers by *BA* supplementation in diet (Teng et al., 2017). In this study, although no positive effect of all treatments was noted on growth, the intestinal microflora was significantly affected. Supplementation with BA and FBSFP at 0.25 % and 0.5 % in the diet significantly increased the lactic acid bacterial counts in the ileum of chickens (Table 3). In contrast to the antibacterial effects observed in the in vitro test, dietary supplementation with FBSFP did not significantly reduce the *E. coli* count in the ileum and cecum of chickens. However, *BA* supplementation showed a significant reduction in *E. coli* in the ileum. A possible explanation is that, within the intestinal environment, the competitive inhibition effect of *BA* may not exert an additive interaction with the antibacterial activity of FBSFP against *E. coli*. Other studies have also reported the positive effect of *BA* on the ileal villus height. Increased villus height suggests enhanced intestinal surface area, contributing to better nutrient adsorption (Xu et al., 2002). Furthermore, deeper crypts indicate faster cell turnover to meet the needs of new tissues responding to inflammation or molting caused by bacterial toxins or pathogens (Yason et al., 1987). This study noted a similar effect with the FBSFP group showing a higher villus height/crypt depth ratio in the jejunum compared to the control group (Table 4). In this study, the FBSFP and *BA* groups did not show an increase in villus height or crypt depth, which may have limited the enhancement of intestinal nutrient absorption surface area, thereby resulting in no improvement in growth performance.

In recent years, oxidative stress has become a crucial issue for the commercial poultry industry (Li et al., 2024; Lee et al., 2023; Tsai et al., 2022). Genetic selection of leanness, larger breast muscles, and rapid growth rates make poultry, such as broilers, particularly vulnerable to oxidative stress (Estévez, 2015). In animals, oxidative stress is an important mechanism leading to biological damage, several pathological conditions, and growth inhibition (Fellenberg and Speisky, 2006). The reduction of free molecular oxygen results in the generation of reactive oxygen species (ROS) such as superoxide anions (O_2_^-^), hydrogen peroxide (H_2_O_2_), and hydroxyl radicals (OH^-^). These free oxygen radicals cause peroxidative decomposition of phospholipids, and further lead to the accumulation of MDA (Bhutia et al., 2011). ROS accumulation is regulated *in vivo* by antioxidant enzymes such as SOD, CAT, and GPx (Aliahmat et al., 2012). However, this study showed that supplementation of *BA*, 0.5 % BSFP, and FBSFP (0.25 % and 0.5 %) in diets significantly decreased the serum MDA concentration (Table 5). It is also worth noting that 0.5 % FBSFP supplementation in diets could increase serum CAT and GSH-Px activities. The above findings suggest that the addition of *BA*, BSFP, and FBSFP could enhance the antioxidant level of chickens.

Fig. 6 shows the results of several serum parameters, according to which supplementation of *BA* could decrease CHOL levels in serum. Pereira and Gibson (2002) indicated that cholesterol in the intestinal tract of animals could be incorporated into the cell membrane of *Lactobacillus*. According to the above-discussed results, *BA* may increase lactic acid bacteria in the intestine and may be the possible reason for the decline in serum cholesterol in chickens. On the other hand, serum TNF-α concentration was also determined as an indicator of immunomodulatory effect. Several pro-inflammatory cytokines, including TNF-α, interleukin-1 (IL-1), and IL-6, are excreted by monocytes and macrophages. These cytokines are involved in the inflammatory response by activating neutrophils, monocytes, and macrophages to stimulate the proliferation of T and B lymphocytes, initiating bacterial and tumor cell killing. These cytokines are also associated with systemic inflammation, such as fever and liver protein synthesis (Yang et al., 2008). The results of this study showed that FBSFP supplementation at both doses significantly increased serum TNF-α levels. COS containing chitihexaose has been reported to stimulate the release of TNF-α from macrophages (Zhang et al., 2014). This effect may be due to the presence of COS in the FBSFP, as COS-containing chitihexaose might be the possible reason for the increase in the effect of serum TNF-α.

However, the changes in growth parameters induced by probiotics and other feed additives may also influence changes in meat quality (Chen et al., 2020; Popova, 2017). Feeding strategies using probiotics show potential as an efficient approach to improve poultry meat quality. Therefore, the meat quality characteristics were also analyzed in this study. Consumers tend to prefer meat with low-brightness, high-redness, and low-yellowness (Wei et al., 2017). This study found that supplementation of 0.25 % and 0.5 % FBSFP in diets could decrease the L* (lightness) and the b* (yellowness) of chicken breast muscle (Table 8). This finding is similar to that reported by Wei et al. (2017) who administered *BA* in broiler diets. The color of meat is affected by heme and pigment content. The observed changes in meat color may be related to the influence of probiotics on muscle metabolic processes and the enhancement of antioxidant capacity, which could affect myoglobin stability and oxidative status in muscle tissue. However, the exact mechanism through which *BA* and FBSFP supplementation affect meat color is yet to be further explored.

In the poultry industry, ammonia, indole compounds, and sulfur compounds are among the most important undesirable gases that emit odor and cause severe air pollution. Researchers suggest that fecal odor and NH_3_ emissions are related to nutrient utilization efficiency and the balance of the intestinal microflora (Teng et al., 2017; Ferket et al., 2002). The previous studies reported that supplementing probiotics in diets can indirectly reduce these environmental pollutants in animal feces by improving intestinal microbiota composition and nutrient utility (Ferket et al., 2002). In this study study, *BA* and FBSFP supplementation led to a significant decline in *E. coli* count, indole content, and skatole (3-methylindole) content in litters (Table 9). In fact, research on chicken shows that *Bacillus* species can inhibit the growth of pathogenic microorganisms and reduce ammonia emissions (Chen et al., 2012; Lei et al., 2014). The current results indicate that *BA* can also effectively inhibit ammonia production.

## Conclusion

This study confirmed that that the combined functionality of *BA* and COS, supplementation of 0.5 % FBSFP could effectively improve the balance of intestinal microbiota, intestinal morphology, antioxidant levels, and partial meat quality of female Red Feather Native chicken. FBSFP is also an effective feed additive to inhibit odor production in chicken husbandry. According to the results of this study, FBSFP shows great potential as a functional feed additive for chickens.

## Author contributions

Conceptualization_Che Lun Chang and Tzu Tai Lee; Data curation_Che Lun Chang; Formal analysis_Che Lun Chang and Shen Chang Chang; Funding acquisition_Tzu Tai Lee; Investigation_Min Jung Lin; Methodology_Li Jen Lin; Project administration_Tzu Tai Lee; Resources_Tzu Tai Lee; Software_Che Lun Chang and Shen Chang Chang; Supervision_Tzu Tai Lee; Validation_Li Jen Lin; Visualization_Tzu Tai Lee; Writing - original draft_Tzu Tai Lee; Writing - review & editing_Tzu Tai Lee.

## Data availability

The data presented in this study are available on request from the corresponding author upon reasonable request.

## Disclosures

We declare that there is no conflict of interest with any financial organization regarding the material discussed in the manuscript.
